# Laparoscopic-thoracoscopic esophageal resection in the treatment of giant epiphrenic esophageal diverticulum (Ivor Lewis operation): Case report

**DOI:** 10.1016/j.ijscr.2018.10.005

**Published:** 2018-10-08

**Authors:** Аlexander Khitaryan, Anastasiya Golovina, Arut Mezhunts, Kamil Veliev, Raisa Zavgorodnyaya, Аlexey Orekhov

**Affiliations:** a NGHCI Railway Clinical Hospital at the “Rostov-Glavnyy” Station, OAO Russian Railways, Varfolomeeva Street 92, Rostov-on-Don, Russian Federation^1^; b FSBEI HE Rostov State Medical University of the Ministry of Health of the Russian Federation, Nakhichevansky Lane 19, Rostov-on-Don, Russian Federation^2^

**Keywords:** Esophageal diverticulum, Epiphrenic diverticulum, Laparoscopic esophageal surgery, Necrotic diverticulitis, Laparoscopic-thoracoscopic esophageal surgery, Case report

## Abstract

**Introduction:**

Complicated esophageal diverticulum is an extremely rare disease. The most widespread surgical tactic in such cases is drainage of the mediastinum and the position of a feeding gastrostomy or enterostomy. Our objective was to demonstrate the possibility of performing a one-step operation in case of giant esophageal diverticulum with necrotic diverticulitis and sepsis on the background of dilatation of the esophagus.

**Presentation of case:**

A 57-year-old woman complained of persistent nagging epigastric pain, heartburn, belching, daily vomiting of eaten food and liquid, impaired swallowing, fever up to 39° C. EGD revealed large pouch of the right esophageal wall in middle and lower third of the esophagus with signs of inflammation, ulceration and necrosis and retained food in the pouch. Barium esophagogram demonstrated 50 × 100 mm epiphrenic diverticulum on the right side and in the lower third of the esophagus which contained food. CBC revealed leukocytosis along with “left upper shift” and ESR.

**Discussion:**

The laparoscopic-thoracoscopic esophageal resection with gastric tube plasty (Ivor Lewis operation) might be the method of choice in cases of giant epiphrenic esophageal diverticula, when esophageal resection is connected with the high risk of anastomotic dehiscence due to esophageal wall necrosis.

**Conclusion:**

We have established that in case of necrobiotic changes in the esophageal wall in the area of the diverticulum, this tactic is completely justified because of the greater patient safety and the less risk of the stapler suture line dehiscence.

## Highlights

• Complicated epiphrenic diverticula are extremely rare.
• A 57-year-old woman had giant diverticulum with spread esophageal wall necrosis.
• One-step laparoscopic-thoracoscopic esophageal resection with gastric tube plasty.
• Treatment of giant diverticulum with necrotic diverticulitis and sepsis on the background of dilatation of the esophagus.
• The patient had an uneventful recovery and was discharged 11 days after the surgery.

## 1 Introduction

Epiphrenic diverticula (ED) are mostly acquired lesions that usually have pulsion etiology. According to the literature, their prevalence is 10–15% of all esophageal diverticula [1,2]. There is an estimated prevalence of 0.015% in the United States, 0.77% in Japan and up to 2% in Europe, but the true incidence is unknown as only small percentage of patients are symptomatic [3,4]. The etiology and pathogenesis of ED are not fully revealed [5,6]. According to Brandeis A.E. and co-authors, the frequency of epiphrenic diverticula complications ranges from 3.5% to 5.7% [7].

A complicated esophageal diverticulum is an extremely rare disease. A perforation in the area of the diverticulum, associated with inflammation after ingestion of a foreign body or rupture of the esophagus against the background of diverticulitis after alcohol intake and diet violation can be a morphological substrate of complications. Such clinical situation can cause sepsis and mediastinitis. Traditional surgical tactic, in this case, is drainage of the mediastinum and the position of a feeding gastrostomy or enterostomy. The development of endoscopic technologies allows replacing stoma with endoscopic stenting with the use of covered esophageal stents in this situation. The disadvantages of stenting are short stent length in the presence of dilated esophagus over a large extent and the necessity for a two-stage treatment of the patient.

Our objective was to demonstrate the possibility of performing a one-step in the treatment of the patient with giant esophageal diverticulum with necrotic diverticulitis and sepsis on the background of dilatation of the esophagus. The operation was performed in the surgical department of the non-governmental clinical hospital.

The work has been reported in line with the SCARE criteria [8].

## 2 Presentation of case

A 57-year-old Caucasian woman was admitted to our surgical department. She complained of persistent nagging epigastric pain and pain behind the sternum, in the left hypochondrium, constant heartburn, belching, daily vomiting of eaten food and liquid, impaired swallowing, fever up to 39° C. The woman had no previous medical or surgical history.

**Fig. 1:**
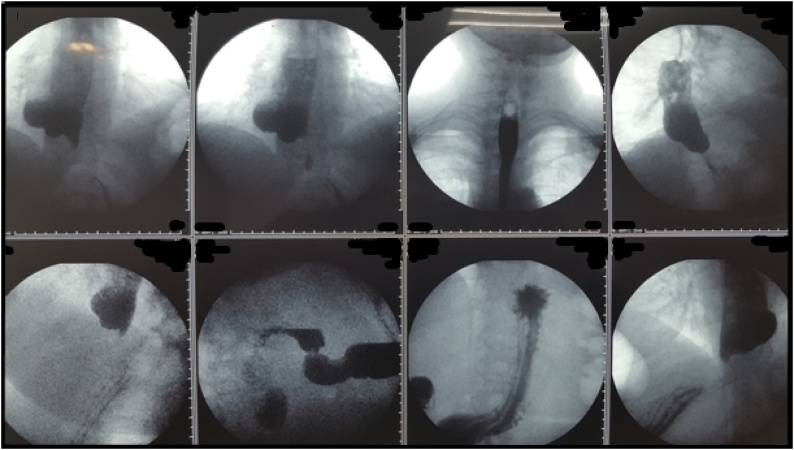
Barium swallow esophagoscopy (see comments in the text).

The preoperative study included esophagogastroduodenoscopy (EGD), barium swallow esophagoscopy and chest spiral computed tomography (CT) scan. EGD revealed large pouch of the right esophageal wall in middle and lower third of the esophagus with signs of inflammation, ulceration and necrosis and retained food in the pouch. Barium esophagogram demonstrated 50 × 100 mm epiphrenic diverticulum on the right side and in the lower third of the esophagus which contained food (Fig. 1). CT scan confirmed the diagnosis, showing 77 × 53 mm diverticulum in the lower third of the esophagus. Complete blood count (CBC) revealed leukocytosis along with “left upper shift” and increased erythrocyte sedimentation rate (ESR).

Considering esophageal wall necrosis extended more than 70 mm, the setting of the esophageal stent was inappropriate. Thereby, it was decided to perform laparoscopic-thoracoscopic esophageal resection (Ivor Lewis operation). The time from the moment of admission to the hospital until the operative treatment was 2 days.

### 2.1 Operative technique

**Fig. 2:**
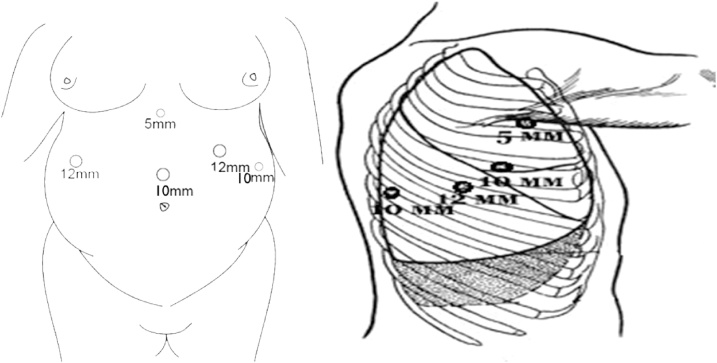
Trocar sites with sizes in mm.

The patient was initially placed in the supine position, and a double lumen endotracheal tube was placed in preparation for the thoracoscopic mobilization of the esophagus. Five abdominal ports were used for the gastric mobilization (see Fig. 2). After an initial inspection of the peritoneal surfaces, the intense intra-abdominal adhesions were revealed and separated. Then the dissection was performed along the greater curvature from the body of the stomach to the subcardial part using bipolar tissue sealer EnSeal (Ethicon US, LLC). The greater omentum was divided from the stomach preserving the right gastric artery and right gastroepiploic artery. The left gastric artery was twice clipped and cut. The left gastroepiploic artery together with short gastric arteries was cut with EnSeal at the point of furcation from the splenic artery. The diaphragmatic cruses were mobilized and partially dissected, expanding esophageal opening of the diaphragm for gastric conduit. The distal segment of the esophagus was mobilized and then cut off using Endopath Echelon Flex 60 linear stapler, with a 60 mm cassette with the guiding ligatures left. The gastric tube as the substitutional conduit was then formed to translocate it into the pleural cavity. A test was made for the blood supply of the stump of the stomach (stomach tube). The blood supply was satisfactory.

**Fig. 3:**
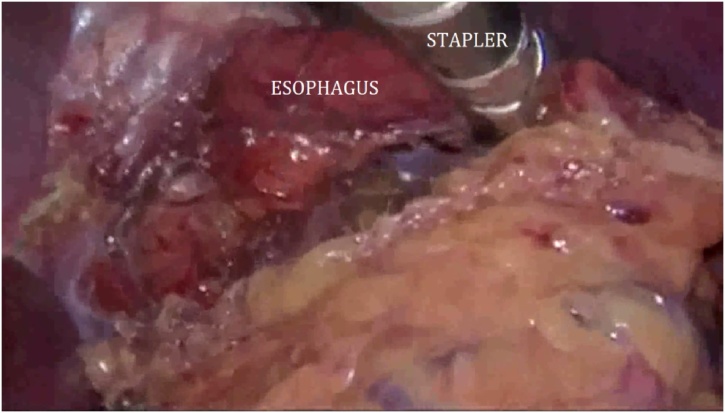
Mobilization of the esophagus.

**Fig. 4:**
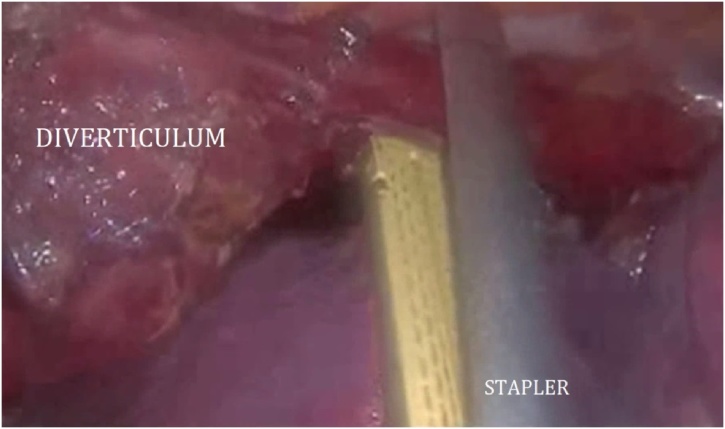
Resection of the esophagus with the diverticulum.

**Fig. 5:**
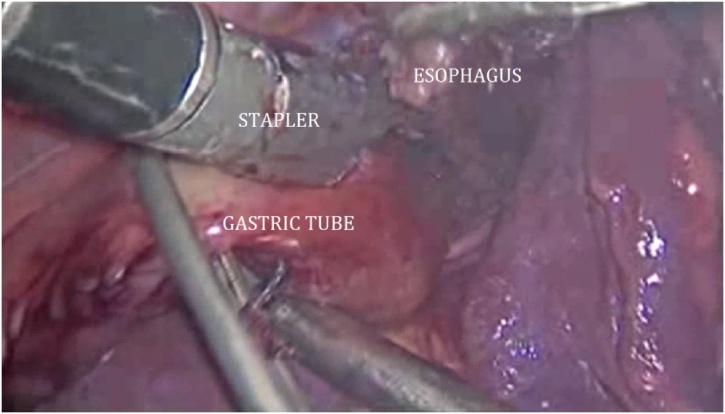
Thoracoscopic stapled esophagogastroanastomosis.

**Fig. 6:**
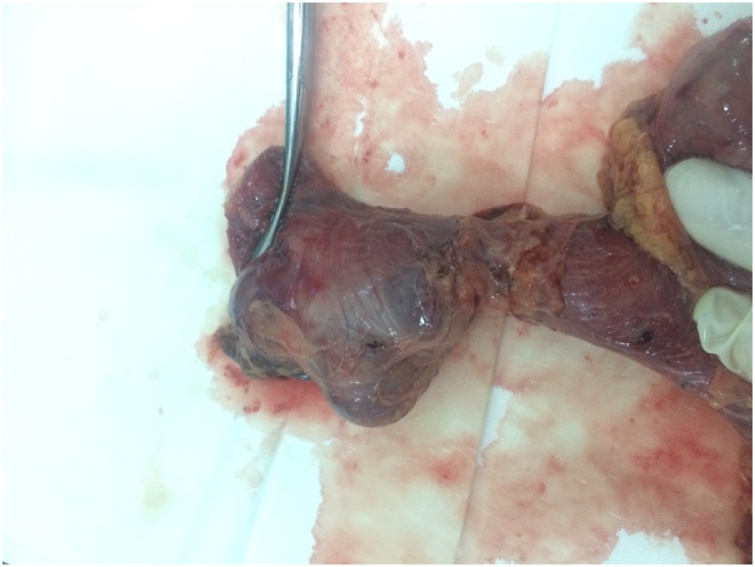
Surgical specimen.

The patient was then turned to the left lateral decubitus position for the thoracoscopic mobilization of the esophagus and creation of the intrathoracic anastomosis. Four trocars were placed into the right pleural cavity (Fig. 2). The esophagus together with giant diverticulum was mobilized up to v.azygos plane (Fig. 3). The paraesophageal lymph nodes were removed. The esophagus was cut using Endopath Echelon Flex 60 linear stapler, with a 60 mm cassette (Fig. 4). Then the gastric tube was pulled into the mediastinum and trial comparison of the edges of the anastomosis without tension was performed. Only after the trial the thoracoscopic stapled esophagogastroanastomosis was performed using the Endopath Echelon Flex 45 stapler, with a 45 mm cassette, closing the anastomosis front wall with a PDS 2-0 suture (Fig. 5). In the next place, the anastomosis zone was covered with stomach with the purpose of antireflux protection. An underwater test of the anastomosis was performed; there was no data for dehiscence and leakage. Then the pleural cavity was drained on the right side by two drain tubes; trocars were removed and trocar wounds were sutured. The resected specimen was sent to the laboratory for histological analysis (Fig. 6).

**Fig. 7:**
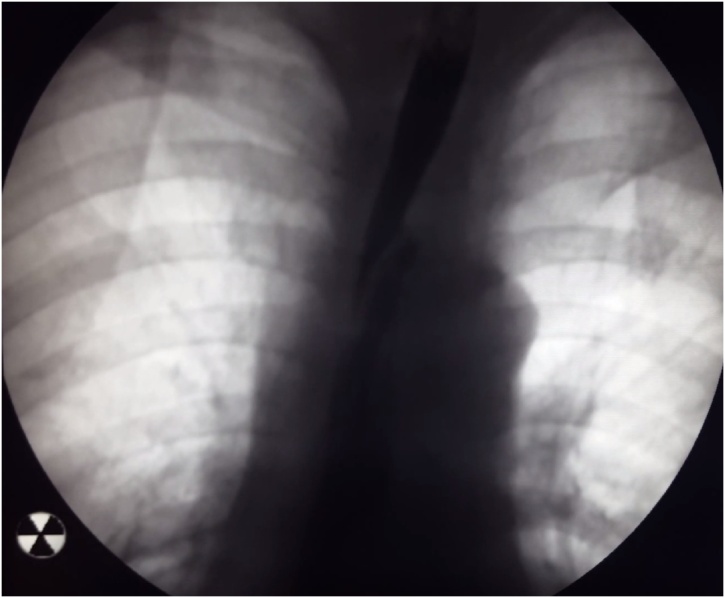
Postoperative esophagogram with Urografin swallow.

The surgery was performed by the team, consisted of 3 experienced in laparoscopy and thoracoscopy surgeons. The overall operative time was 275 min. The patient tolerated the surgery well and was transferred from the intensive care unit to the general hospital room on the next day after the surgery. On the 2nd day a liquid diet was prescribed; it was tolerated without any pain, regurgitation or dysphagia. On the 7th day after the surgery an esophagogram with liquid contrast (Urografin) was performed (Fig. 7).

**Fig. 8:**
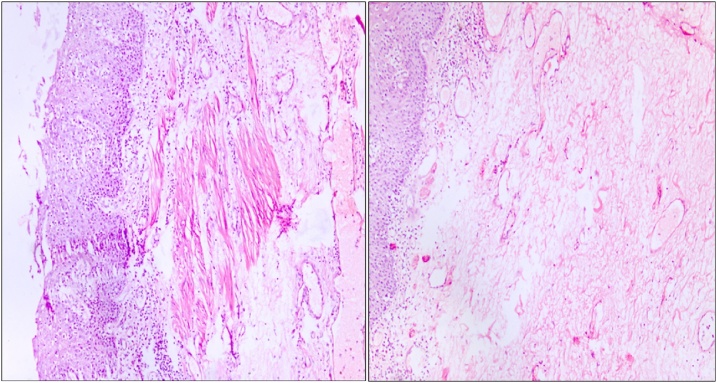
The histological analysis (leukocyte infiltration and focal necrosis).

The results of the histological study revealed the presence of chronic purulent inflammation in the diverticulum wall with focal necrosis of the muscular layer, myxomatosis (Fig. 8).

On the 11th day after the surgery the patient was discharged from hospital in a good state of health with standard dietary and medical recommendations. There were no postoperative complications, no symptoms of dysphagia, nausea or vomiting. The patient was also recommended to perform control EGD 6 months after the operation.

## 3 Discussion

The laparoscopic-thoracoscopic esophageal resection with gastric tube plasty (Ivor Lewis operation) might be the method of choice in case of a giant epiphrenic esophageal diverticulum when esophageal resection is connected with the high risk of anastomotic dehiscence due to esophageal wall necrosis.

The development of endoscopic surgical technique along with the improvement of intracorporeal sutures of digestive anastomoses and the possibility to perform the thoracic stage with a minimally invasive thoracoscopic method allow reducing the traumatic nature of the operation, as well as the risks of postoperative complications significantly [9]. Mastering the technique of endoscopic anastomoses can reduce the amount of blood loss and make the operation duration comparable in time with open access. This fact makes the Ivor-Lewis technique quite attractive treatment option.

In this case the histological examination showed the spread of inflammatory infiltration of the esophagus tissues above the diverticulum. This fact determined the implementation of an esophageal resection to the border of the middle and the lower third.

The minimally invasive operation allowed the patient to activate quickly, thus preventing the development of thrombosis and cardio-respiratory complications.

## 4 Conclusion

We have successfully performed laparoscopic-thoracoscopic esophageal resection (Ivor Lewis operation) in the treatment of the patient with giant epiphrenic esophageal diverticulum. We have established that in case of necrobiotic changes in the esophageal wall in the area of the diverticulum, this tactic is completely justified because of the greater patient safety and the less risk of the stapler suture line dehiscence.

## Conflict of interest

The authors declare no conflicts of interest. The authors have no financial, consultative, institutional and other relationships that might lead to bias or conflict of interest.

## Funding

None.

## Ethical approval

Ethical approval has been exempted by our institution, NGHCI Railway Clinical Hospital at the “Rostov-Glavnyy” station, OAO Russian railways, Rostov-on-Don, Russian Federation.

## Consent

Written informed consent was obtained from the patient for publication of this case report and accompanying images. A copy of the written consent is available for review by the Editor-in-Chief of this journal on request.

## Authors contribution

Khitaryan А. – conceptualization, funding acquisition, investigation, methodology, project administration, resources, supervision, verification, writing original draft, writing review & editing.

Golovina A. – conceptualization, formal analysis, project administration, resources, software, visualization, writing original draft, writing review & editing.

Mezhunts A. – data curation, formal analysis, software, visualization, writing original draft, writing review & editing.

Veliev K. – data curation, investigation, writing original draft, writing review & editing.

Zavgorodnyaya R. – data curation, investigation, writing original draft, writing review & editing.

Orekhov А. – conceptualization, writing original draft, writing review & editing.

## Registration of research studies

None.

This publication is neither ‘first-in-man study’ nor a research.

## Guarantor

Khitaryan А.G.

Golovina A.A.

## Provenance and peer review

Not commissioned, externally peer reviewed.
